# Preliminary evaluation of an analog procedure to assess acceptability of intimate partner violence against women: the Partner Violence Acceptability Movie Task

**DOI:** 10.3389/fpsyg.2015.01567

**Published:** 2015-10-12

**Authors:** Enrique Gracia, Christina M. Rodriguez, Marisol Lila

**Affiliations:** ^1^Department of Social Psychology, University of Valencia, Valencia, Spain; ^2^Department of Psychology, University of Alabama at Birmingham, Birmingham, USA

**Keywords:** acceptability, analog tasks, attitudes, implicit measures, intimate partner violence

## Abstract

Acceptability of partner violence against women is a risk factor linked to its perpetration, and to public, professionals’ and victims’ responses to this behavior. Research on the acceptability of violence in intimate partner relationships is, however, limited by reliance solely on self-reports that often provide distorted or socially desirable accounts that may misrepresent respondents’ attitudes. This study presents data on the development and initial validation of a new analog task assessing respondents’ acceptability of physical violence toward women in intimate relationships: the Partner Violence Acceptability Movie Task (PVAM). This new analog task is intended to provide a more implicit measure of the acceptability of partner violence against women. For this analog task, clips were extracted from commercially available films (90-s segments) portraying partner violence. Two independent samples were used to develop and evaluate the PVAM: a sample of 245 undergraduate students and a sample of 94 male intimate partner violence offenders. This new analog task demonstrated acceptable internal consistency. Results also indicated adequate construct validity. Both perpetrators and undergraduates scoring high in the PVAM also scored higher in self-reported justifications of partner abuse. Perpetrators of partner violence scored significantly higher in acceptability of partner violence than the undergraduate sample (both male and female students), and male students scored higher than females. These preliminary results suggest that the PVAM may be a promising tool to assess the acceptability of violence in intimate partner relationships, highlighting the need to consider alternatives to self-report to evaluate potential beliefs about partner violence.

## Introduction

Intimate partner violence against women by their male partners (IPVAW) is a widespread phenomenon with profound consequences for women’s physical, psychological and social well-being, as well as for the wider society (Campbell, 2002; Ellsberg et al., 2008; World Health Organization (WHO), 2013). With an estimated global prevalence of 30% (23.2% in high-income regions), and a global percentage of female homicides committed by their intimate partners of 38.6% (41.2% in western countries), IPVAW is considered the most common form of violence suffered by women (Devries et al., 2013; Stöckl et al., 2013; World Health Organization (WHO), 2013). Violence against women is typically committed by their male partners, for virtually every form of violence (Hamby, 2014). For example, the main risk of homicide for a woman is from an intimate partner (primarily men), with the proportion of women killed by a partner six times the proportion of men killed by female partners (Stöckl et al., 2013).

A recent survey among the 28 European Union Member States estimated that an average of 22% of European women have been victims of physical and/or sexual violence by their partners since the age of 15, with a prevalence across countries ranging from 13 to 32% (European Union Agency for Fundamental Rights, 2014). According to this survey, the lifetime prevalence of IPVAW in Spain, where the present study was conducted, is 13%, the lowest in the European Union. Regarding public attitudes toward IPVAW, another European survey indicated that victim-blaming attitudes are still widespread across countries, with an average of 55% of European citizens considering that “provocative” behavior in women is a cause of domestic violence (European Commission, 2010). Again, compared to other European countries, the prevalence of these attitudes in Spain was 33%, the lowest for the European Union (which ranged from 33 to 86%).

Attitudes of tolerance and acceptability of IPVAW are still widespread and have been increasingly considered a central issue to understand individual and social factors that contribute to its prevalence in society, thereby representing a main target for intervention and public health efforts (World Health Organization (WHO), 2002; Flood and Pease, 2009; García-Moreno et al., 2014). Similarly, attitudes of tolerance and acceptability of IPVAW are known risk factors linked to its perpetration (Sugarman and Frankel, 1996; Cauffman et al., 2000; Archer and Graham-Kevan, 2003; Bryant and Spencer, 2003). These attitudes have also been linked to public perceptions and responses to IPVAW (West and Wandrei, 2002; Taylor and Sorenson, 2005; Gracia and Herrero, 2006; Frye, 2007), as well as the response of professionals (Home, 1994; Logan et al., 2006; Gracia et al., 2014) and victims toward IPVAW (Barnett, 2001; Rizo and Macy, 2011).

Acceptability of IPVAW is also related to the kind of behavior that is considered violence in intimate relationships. For example, IPVAW may be seen as more acceptable in certain circumstances (e.g., when victims are blamed or considered responsible for provoking violence), or as long as some limits of severity are not crossed, judging IPVAW unacceptable largely in extreme and severe cases (Cauffman et al., 2000; Jewkes, 2002; Worden and Carlson, 2005; Gracia, 2014). If some IPVAW incidents are perceived as less serious, more acceptable, or even deserved in certain circumstances, this may lead to the persistence of this type of behavior among perpetrators and to its justification by the victims themselves and their social circle (Taylor and Sorenson, 2005; Waltermaurer, 2012). This situation may also inhibit victims from disclosing the violence, seeking support, or leaving the relationship, as they may believe their social circle accepts this violence or considers it justified, therefore leading victims to consider help as less likely or deserved (Flood and Pease, 2009; Kogut, 2011).

Adequate measures to assess attitudes toward IPVAW are thus important research and intervention tools as they are key to better understanding its prevalence among clinical and non-clinical samples, to explore its correlates, and to evaluate outcomes or monitor changes after clinical interventions or public health efforts (Flood and Pease, 2009; Eckhardt et al., 2012; Lila et al., 2014a). Adequate assessment of attitudes toward IPVAW is, however, a challenging issue. Research on the acceptability of violence in intimate partner relationships, particularly among clinical samples, is limited by reliance on self-reports that often provide accounts that may misrepresent respondents’ attitudes, distorted by respondents’ self-deception or social desirability biases (Eckhardt et al., 2012).

Direct self-reports can be viewed as an explicit assessment of attitudes, which are particularly vulnerable to participants’ distortion (Fazio and Olson, 2003). In sensitive areas, like partner or family violence (child abuse, partner violence, dating violence, etc.), respondents may avoid sharing their beliefs because of a fear of negative consequences or judgments (Bennett et al., 2006), leading to a distorted response style, providing inaccurate information or presenting themselves, consciously or unconsciously, in a socially acceptable manner (Eckhardt and Dye, 2000; Rodriguez et al., 2011; Eckhardt et al., 2012).

The connection between implicit and explicit attitudes has been articulated in the dual-attitude hypothesis, wherein attitudes include an explicit component and an automatically activated, implicit component (Wilson et al., 2000). People convey their attitudes in part based on whether they have sufficient cognitive resources to access their explicit attitude and override their implicit attitude (Wilson et al., 2000). Reaction time has been utilized in analog tasks to assess such implicit attitudes given that slower reactions are evident when more cognitive capacity is required to derive a response. For example, when presented with information that is consistent with implicitly held beliefs, the attitude is easier and faster to recognize, but it is harder and slower to access when presented with something inconsistent with the implicit attitude, as observed in the Implicit Association Test (IAT; Greenwald et al., 1998), a widely used implicit approach to measuring an array of attitudes.

Although self-reports on sensitive issues might be more reliable and accurate for general population or community samples when, for example, anonymity and confidentiality are ensured (Bowling, 2005; Hamby, 2014), the issue of response distortion is quite problematic with clinical samples like IPVAW offenders (Heckert and Gondolf, 2000; Henning et al., 2005; Scott and Straus, 2007; Eckhardt et al., 2012). As Eckhardt et al. (2012) noted, self-report measures to assess violence-related attitudes among IPVAW offenders are limited by their tendency to defend themselves, to deny or minimize their violent behavior, and to disguise their inclination to use violence (Eckhardt and Dye, 2000; Heckert and Gondolf, 2000; Henning et al., 2005; Henning and Holdford, 2006). According to Eckhardt et al. (2012), “the exclusive use of explicit measures of cognitive constructs has limited the complete understanding of the role played by attitudinal factors in the etiology of IPV. IPV models will be more comprehensive and results of treatment effectiveness research more accurate if cognitive assessment methods capture both explicit as well as implicit cognitive processing” (p. 473).

As self-reports regarding attitudes of acceptability of IPVAW may be biased in clinical samples (e.g., IPVAW perpetrators), it is important to explore these attitudes with alternatives such as indirect or implicit measures (Fazio and Olson, 2003; Eckhardt et al., 2012). In contexts where the assessment of attitudes toward violence may be threatened by perpetrators’ denial or minimization, explicit measurements should be complemented by implicit measures (Eckhardt et al., 2012). Indeed, some have suggested that both explicit and implicit measures need to be utilized to ensure that attitude change is not simply in explicit attitudes (Wilson et al., 2000).

Analog procedures measure constructs of interest using indirect, implicit means, attempting to approximate behavior in a manner analogous to the target construct (DeGarmo et al., 2006). In the area of IPVAW, few alternatives to self-reports are available. As one exception, the Implicit Association Test (IAT; Greenwald et al., 1998), has been adapted to assess partner violence attitudes; this IAT did differentiate between a small sample of IPVAW offenders from non-offenders, although IAT scores were not related to self-reported justification of IPVAW (Eckhardt et al., 2012). In the present study, we propose an alternative analog procedure based instead on reaction time delay to video clips to measure the acceptability of IPVAW.

Some video-based analog tasks are available in the area of child maltreatment (Fagot, 1992; Rodriguez et al., 2011), an area that shares characteristics to IPVAW regarding potential response biases. As far as we know, however, no video-based analog tasks assessing attitudes in the area of partner violence are available. Based on the work of Rodriguez et al. (2011) with an analog task on acceptability of parent–child aggression, this study presents data on the development and initial validation of a new analog task assessing attitudes of acceptability of physical violence against women in intimate partner relationships. This study evaluates the Partner Violence Acceptability Movie Task (PVAM), an analog procedure based on responses to video clips depicting physical aggression toward women. Using two samples, the investigation expected that slow responding on the analog task would be associated with self-reported justification of violence toward women. In addition, male IPVAW offenders were expected to react more slowly to video scenes of violence than either male or female college undergraduates.

## Materials and Methods

### Participants

Two independent samples were used to develop and evaluate the PVAM. Sample 1 participants included 245 undergraduate psychology students (189 females and 55 males) enrolled at the University of Valencia. The students’ mean age was 24.47 years (SD = 6.99), and their median family household income was between 18 and 24,000€. Sample 2 included 94 male intimate partner violence perpetrators who were court-mandated to a batterer intervention program (these offenders had been sentenced to less than 2 years in prison and had no previous criminal record, so they received a suspended sentence conditioned on their attendance in an IPVAW intervention program). Offenders’ mean age was 40.80 years (SD = 10.67); median family household income was between 6 and 12,000€. About half of the offenders participated at the start of their treatment (*n* = 48) and the remainder at the conclusion of treatment (*n* = 46).

### Measures

#### Partner Violence Acceptability Movie Task

Created for a larger study of analog tasks, this movie video based analog procedure is designed to measure acceptability of IPVAW which could be less susceptible to social desirability responses than explicit self-reports. PVAM was created following Rodriguez et al. (2011) procedures. First, numerous commercially available films were pre-screened to identify IPVAW scenes that could be used for measure construction (all films considered were films originally in English but were required to be dubbed in Spanish, not subtitled). Clips were selected for inclusion if a 90-s segment could be identified that demonstrated male physical aggression toward the female partner in a relatively continuous scene, occurred early enough in the scene without moving to a different event, and represented the target male and female partners throughout the segment.

Nine clips were initially extracted from commercially available films (90-s segments) showing physical IPVAW from the following films: “*What’s Love Got to Do with It*” (man slapping and punching his partner after she had criticized his work); “*Joy Luck Club*” (man pushing his partner to the ground when she resists another woman being offered to hold her baby); “*Color Purple*” (woman slapped by partner while reading a letter); “*Fried Green Tomatoes*” (man slapping and kicking his wife down stairs because she is leaving him); “*Sleeping with the Enemy*” (man slaps his wife, accusing her of flirting with a neighbor); “*Not without My Daughter*” (man slaps his wife when she tells him she wants them to move out of the country); “*Enough*” (man punches his wife after she accuses him of being unfaithful); “*Klansman*” (man hits his wife when she admits in public she has been unfaithful); and “*Godfather*” (man hits his wife with a belt during an argument after she breaks and throws objects). Clips are presented to participants in random order.

To generate a score reflecting acceptability of IPVAW, participants were asked, based solely on the scene, to stop the video if they consider the man has become too violent. The implicitly assessed score of interest is the amount of time lapsed (measured in fractions of seconds) from the initial physical aggressive contact between the couple until the participant stops the clip. The PVAM total score is derived from the mean delay across film clips. Slower response time in judging a scene as abusive is thus conceptualized as indicating greater acceptability of IPVAW because slower latency in responding implies greater cognitive processing occurred to identify a scene as abusive compared to quick, easily accessed beliefs.

#### Inventory of Beliefs About Wife Beating (Saunders et al., 1987)

This scale measures attitudes and beliefs about wife beating on a 7-point Likert scale, ranging from strongly agree (1) to strongly disagree (7). The measure was translated into Spanish for this investigation. In this study, we used the inventory of beliefs about wife beating (IBWB) Justification scale, a 12-item subscale assessing wife-beating justification (e.g., “There is no excuse for a man beating his wife”). Scores were oriented such that higher scores indicate greater justification of IPVAW. For the Spanish version used in the present study, Cronbach’s alpha was 0.72 (students) and 0.83 (offenders).

### Procedure

Intimate partner violence offenders and undergraduate students participated voluntarily in a larger study of analog tasks. Undergraduate students received coursework credits in exchange for participation. In both samples, after providing informed consent, participants were led to a computer room of the university. First, they completed demographic information immediately followed by the PVAM. Then, they responded to other self-report measures about child-rearing, with the IBWB last in the larger study. All measures were administered via computer and took about 45 min. To ensure anonymity for students, data were coded by random identification numbers never connected to participants’ personal identities; offenders’ responses were also coded by random number and responses were kept confidential. The University of Valencia Ethics Committee approved the study.

## Results

### Preliminary Review of PVAM Items and Internal Consistency

To check that participants were not mistakenly reacting too quickly and were complying with the task, all individual scores were scanned for responses that terminated the scenes before any physical contact had occurred between the depicted perpetrator and victim in the scene. As a result of this validity check, two films were removed from the final scores, *Klansman* and *Godfather*, because more than 25% of respondents appeared confused by these scenes and terminated early (compared to less than 2% on other films). Thus, the resulting PVAM scores are based on the remaining seven videos. To be conservative, participants that had questionable reaction times for other videos were removed, resulting in the removal of nine perpetrators’ PVAM scores in Sample 2 and two female undergraduate students in Sample 1. In terms of reliability for the seven videos contributing to the PVAM average score, alpha was 0.72 for students and 0.81 for IPVAW offenders.

### Correlational Findings

The IBWB Justified Scale scores were significantly positively correlated with average PVAM scores for both samples: Students, *r* = 0.25, *p* ≤ 0.001; Offenders, *r* = 0.34, *p* ≤ 0.001, indicating greater self-reported justification of violence against women was related to slower termination of videos. The magnitude of these associations was not affected for either sample controlling for participants’ age or income level.

### Sample/Subsample Differences

Obtained scores also differed within and between samples. In Sample 1, for the self-report IBWB Justified Scale, male students obtained significantly higher scores (*M* = 14.62, SD = 4.60) than female students [*M* = 13.42, SD = 3.13; *t*(242) = 2.22, *p* = 0.03, Cohen’s *d* = 0.31]. Similarly, on the PVAM average scores, male students obtained significantly higher (slower termination) scores (*M* = 7.57, SD = 6.55) than female students [*M* = 5.60, SD = 4.49; *t*(240) = 2.38, *p* = 0.02, *d* = 0.35]. Offenders in Sample 2 also obtained slower PVAM scores (*M* = 8.90, SD = 10.18) than either male students [*t*(84) = 2.13, *p* = 0.03, *d* = 0.16] or female students [*t*(84) = 3.54, *p* = 0.00, *d* = 0.42]. Similarly, offenders obtained higher IBWB Justified Scale scores (*M* = 18.60, SD = 7.41) than both female [*t*(89) = 6.63, *p* = 0.00, *d* = 0.91] and male students [*t*(89) = 5.09, *p* = 0.00, *d* = 0.65]. Within the offenders sample, those men who were at the start of treatment obtained comparable scores on the IBWB Justified Scale (*M* = 18.99, SD = 7.57) to those at the end of treatment [*M* = 18.17, SD = 7.29, *t*(88) = 0.53, *p* = 0.60, *d* = 0.11]. In contrast, men in Sample 2 who were at the end of treatment responded more quickly on the PVAM (*M* = 6.29, SD = 4.58) than men at the start of treatment [*M* = 11.12, SD = 12.84; *t*(83) = 2.23, *p* = 0.02, *d* = 0.50; see Table 1].

**TABLE 1:** **Sample/subsample differences in IPVAW scores**.

	***M* (SD)**	***t*-test**
**PVAM**		
Female students	5.60 (4.49)	^a^3.54^***^
Male students	7.57 (6.55)	^a^2.13^*^
Offenders (overall)	8.90 (10.18)	
Pre-treatment	11.12 (12.84)	^b^2.23^*^
Post-treatment	6.29 (4.58)	
**IBWB**		
Female students	13.42 (3.13)	^a^6.63^***^
Male students	14.62 (4.60)	^a^5.09^***^
Offenders (overall)	18.60 (7.41)	
Pre-treatment	18.99 (7.57)	^b^0.53
Post-treatment	18.17 (7.29)	

^a^Comparison with offenders overall. ^b^Comparison between pre-post treatment. ^*^p ≤ 0.05, ^***^p ≤ 0.001.

## Discussion

In this paper, we described the development and psychometric properties of the PVAM as a preliminary evaluation of this new video-based analog task assessing acceptability of IPVAW. This new procedure, based on the assessment of reaction time in response to video clips showing male physical aggression toward female partners, aimed to provide an analog measure of the acceptability of IPVAW. Data from two samples, including male and female undergraduate students, and male perpetrators of IPVAW, provided evidence of adequate internal consistency, as well as construct validity. In general, these preliminary results suggest that this new analog task can provide a psychometrically sound and valid procedure to assess acceptability of IPVAW, providing a complimentary tool to self-reports evaluating attitudes and beliefs regarding partner violence.

Correlations and mean comparisons between samples supported the construct validity of the new analog task, as they provided evidence for both content relevance and representativeness, and criterion-relatedness (Messick, 1995). Regarding content relevance, correlations between PVAM scores and IBWB Justified Scale self-reports, two measures of closely related constructs (i.e., acceptability of IPVAW and wife-beating justification), were significant for both student and offender samples. Results indicate that those respondents that took longer to stop the videos, indicating greater IPVAW acceptability, were also those who reported greater wife-beating justification. Correlations between the analog task and the self-report measure were, however, of mild to moderate magnitude (Cohen, 1988), which is expected given the moderately explicit nature of the PVAM analog task (Fazio and Olson, 2003). Although respondents to the PVAM provide direct responses to scenes depicting partner aggression, they are less likely to realize that the task is actually assessing the “degree” of their acceptability of these types of behaviors via their response time scores.

Regarding criterion-relatedness of the new analog task, several findings in this study also support its construct validity. Results from mean comparisons showed that the new analog task differentiated between and within samples as would be expected. In our study, perpetrators and students were differentiated on the basis of both explicit and the more implicit PVAM measure, which contrasts with other studies that failed to find differences between offenders and non-offenders on the basis of explicit self-report on the IBWB (e.g., Eckhardt et al., 2012). Perhaps the nature of our comparison group (students in our sample versus male non-offenders from the community in Eckhardt et al., 2012) accounts for our different result on self-report. Offenders in our sample scored slower on the PVAM task than students (either male and female), indicating the former’s greater acceptability of IPVAW. Similarly, IPVAW perpetrators showed greater self-reported justification of violence against women than students. Both results support the link between attitudes toward IPVAW and its perpetration (Sugarman and Frankel, 1996; Cauffman et al., 2000; Archer and Graham-Kevan, 2003; Bryant and Spencer, 2003). Both self-report and analog task measures also differentiated male and female students. According to Flood and Pease (2009) review, gender is one of the more consistent predictors of attitudes and beliefs supporting the use of IPVAW. Our results support this view, as male students showed greater self-reported justification of wife beating and greater acceptability of IPVAW (as indicated by their slower responses to videos) than female students.

Regarding criterion-related evidence, results in the present study suggest that the new analog task was a more sensitive measure to detect change among perpetrators participating in a batterer intervention program than the self-report. There were no differences between perpetrators at the beginning and at the end of the intervention program in self-reported justification of IPVAW. The PVAM, however, distinguished between those offenders at the beginning of the intervention program (with slower response time to stopping the video aggression) from those who had concluded the intervention program. Perpetrators finishing the intervention program responded more quickly to the videos suggesting lower acceptability of IPVAW among these offenders. Although these results should be considered preliminary and exploratory, as they are not based on an experimental design supporting the actual efficacy of the intervention program, nevertheless we believe that this finding is a particularly interesting one in terms of a potential indicator of change or program efficacy (Scott, 2004; Lee et al., 2007; Scott et al., 2011; Lila et al., 2014b). The use of more implicit measures to assess attitudes in batterer intervention program (which may be more sensitive than self-reported measures that are more susceptible to concealment and social desirable reporting), may provide new assessment approach for treatment providers. Analog tools may identify, for example, more responsive versus more treatment-resistant offenders, detect different treatment departure points, more accurately track cognitive and attitude changes, and thereby adjust intervention programs accordingly. Also, the availability of more implicit measures in batterer intervention programs may provide new means to strengthen the validity of program evaluation, as accurate assessments of relevant program outcomes such as cognitive and attitudinal changes may be less undermined by offenders’ reporting biases (Chereji et al., 2012; Eckhardt et al., 2012; Arias et al., 2013). Finally, the PVAM provides an alternative to other implicit measures such as the IAT (Eckhardt et al., 2012), as reaction time is less language-dependent and can overcome limitations in IAT implicit measures such as the influence of cognitive inertia or lack of cognitive capability (Messner and Vosgerau, 2010), which may be a particularly relevant issue with clinical samples like IPVAW offenders (Teichner et al., 2001; Babcock et al., 2008; Romero-Martínez et al., 2013).

Some methodological considerations regarding this study deserve some attention. With regard to PVAM performance among offenders, nearly 10% evidenced responses that were technically too fast to be accurate readings (a comparatively unusual pattern among undergraduates). These responses were typically termination of the video before there had even been interaction between the characters in the scene, suggesting non-compliance. Although the video-based platform was intended to be engaging for participants, these findings implying non-compliance may be particularly problematic for this population—a phenomenon that unfortunately also likely detracts from accurate explicit self-reports. Notably, those who terminated too quickly tended to be among the highest scorers on the IBWB Justified scale, which most explicitly justifies violence toward women. If those expressed IBWB attitudes are accurate, those rapid PVAM responses may be most manifest among those with strong IPVAW attitudes who are more resistant to compliance. In part this reflects that even implicit analog measures, although resistant to participant manipulation, are not impervious to impression management (Gawronski et al., 2007; Eckhardt et al., 2012) or simple non-compliance. Moreover, PVAM scores may reflect other processes, including avoidance of content regarding couple relationships. Thus, although the PVAM may provide information that can complement explicit measures, efforts should continue to advance analog procedures that would be more resistant to non-compliance. For example, some analog measures can be designed with virtually no participant response, such as assessments of empathy or attributions with eye-tracking procedures (e.g., Rodriguez et al., 2012). The development of innovative assessment procedures is needed to more confidently assess important constructs in family violence research. The extent to which any implicit task can successfully control for response distortion is unknown, but analogs are intended to address impression management more than explicit measures. However, the current study did not directly query participants regarding their recognition of the intent or scoring of the task; future research studies could consider investigating participants’ perceptions of the task as well as the extent to which the task could otherwise be affected by social desirability responding.

As for other potential limitations, results could also reflect educational differences between the student and offender samples, as research has linked both perpetration and attitudes condoning IPVAW to socioeconomic status (Heise et al., 1999; Waltermaurer, 2012; Gracia and Tomás, 2014). We are confident, however, that results likely reflect real differences in acceptability and justification of IPVAW as our clinical sample were men actually convicted for this offense. Moreover, controlling for age and income yielded no difference in the magnitude of the observed results. Relatedly, the generalizability of the student sample results to the larger population is also a potential limitation, and in this regard, future research should assess the PVAM using community samples representative of the general population. It is important to stress that the analog procedure presented in this paper is in its preliminary stages of validation, and that the reported correlations and comparisons provided represent only initial data. Clearly, future research would benefit from further validation studies of this procedure by using other self-report measures, testing its stability over time, using observational data (e.g., therapists reports), or exploring “hard” data as correlates of attitude change like IPVAW recidivism data. Results also reflect Spanish attitudes that may not apply to other countries, such as other European or North American samples, given that Spain is a country with comparatively lower rates of IPVAW than other western countries. Clearly, future cross-cultural research with new analog procedures would help to better understand variations in IPVAW both within and between countries as they relate to its acceptability in different societies or cultural groups. Efforts to more accurately gage IPVAW attitudes remain critical in order to advance efforts to curtail IPVAW, a major public health concern.

### Conflict of Interest Statement

The authors declare that the research was conducted in the absence of any commercial or financial relationships that could be construed as a potential conflict of interest.
